# Prediction of Ross River Virus Incidence Using Mosquito Data in Three Cities of Queensland, Australia

**DOI:** 10.3390/biology12111429

**Published:** 2023-11-13

**Authors:** Wei Qian, Elvina Viennet, Kathryn Glass, David Harley, Cameron Hurst

**Affiliations:** 1 School of Public Health, Shanghai University of Traditional Chinese Medicine, Shanghai 201203, China; wei.qian@uqconnect.edu.au; 2 UQ Centre for Clinical Research, The University of Queensland, Herston, QLD 4029, Australia; 3 Strategy and Growth, The Australian Red Cross Lifeblood, Kelvin Grove, QLD 4059, Australia; 4 School of Biomedical Sciences, Queensland University of Technology, Kelvin Grove, QLD 4059, Australia; 5 Research School of Population Health, Australian National University, Acton, ACT 0200, Australia; 6 Molly Wardaguga Research Centre, Charles Darwin University, Brisbane, QLD 4001, Australia; 7 Department of Statistics, QIMR Berghofer Medical Research Institute, Brisbane, QLD 4006, Australia

**Keywords:** Ross River virus, mosquitoes, surveillance, prediction, exposures, lagged effects

## Abstract

**Simple Summary:**

Mosquito abundance data from vector surveillance programs can be used to help predict the incidence of Ross River virus (RRV). Climate, weather, geographical, and socio-economic variables also influence RRV incidence. In this study, we aimed to predict RRV incidence rates in three cities of Queensland, Australia (Brisbane, Redlands, and Mackay) and to assess the utility of mosquito data in prediction. Our findings demonstrated that mosquito abundance was a valuable predictor for RRV incidence in Brisbane and Redlands. The predictive results of Brisbane and Redlands were excellent, while for Mackay its prediction was less satisfactory. This study demonstrated the value of mosquito surveillance data for the prediction of RRV incidence in small geographical areas.

**Abstract:**

Ross River virus (RRV) is the most common mosquito-borne disease in Australia, with Queensland recording high incidence rates (with an annual average incidence rate of 0.05% over the last 20 years). Accurate prediction of RRV incidence is critical for disease management and control. Many factors, including mosquito abundance, climate, weather, geographical factors, and socio-economic indices, can influence the RRV transmission cycle and thus have potential utility as predictors of RRV incidence. We collected mosquito data from the city councils of Brisbane, Redlands, and Mackay in Queensland, together with other meteorological and geographical data. Predictors were selected to build negative binomial generalised linear models for prediction. The models demonstrated excellent performance in Brisbane and Redlands but were less satisfactory in Mackay. Mosquito abundance was selected in the Brisbane model and can improve the predictive performance. Sufficient sample sizes of continuous mosquito data and RRV cases were essential for accurate and effective prediction, highlighting the importance of routine vector surveillance for disease management and control. Our results are consistent with variation in transmission cycles across different cities, and our study demonstrates the usefulness of mosquito surveillance data for predicting RRV incidence within small geographical areas.

## 1. Introduction

Ross River virus (RRV) disease is the most common human arboviral infection in Australia. It is caused by an alphavirus. Queensland is a high-risk area for RRV that reports over 2000 cases annually; which accounts for almost half of all cases in Australia [1]. The transmission of RRV involves vectors (mosquitoes) and reservoir hosts (including marsupials, mammals, and birds) [2], and thus is more complicated than a “human-mosquito-human” transmission cycle (e.g., dengue or malaria). Its transmission can be influenced via climate (e.g., the Southern Oscillation Index, known as the SOI), weather (e.g., rainfall, temperature, humidity, and vapour pressure), geographical (e.g., surface water and vegetation cover), and socio-economic factors [3]. Prediction of RRV incidence can provide valuable information to enhance RRV management and control.

A number of mosquito species (e.g., *Aedes vigilax* and *Culex annulirostris*) are clearly important in transmitting RRV to humans. Isolations of RRV have been obtained from the following mosquito species in Queensland: the freshwater-breeding *Culex annulirostris*, salt water-breeding *Aedes vigilax*, peridomestic freshwater-breeding *Aedes notoscriptus*, *Aedes vittiger*, *Aedes imprimens*, *Aedes kochi*, *Culex gelidus*, *Mansonia septempunctata*, *Verrallina carmenti*, *Verrallina lineata*, *Aedes procax*, brackish water-breeding *Aedes funereus*, saltmarsh *Culex sitiens*, and *Aedes alternans* [4,5].

Many studies have demonstrated a link between increased RRV incidence and an increased abundance of all mosquitoes or of a specific species (*Ae. notoscriptus*, *Cx. annulirostris*, *Ae. vigilax*, and *Aedes camptorhynchus*), usually at a lag of one month [6,7,8,9,10,11,12]. In Woodruff et al. (2006), mosquito counts were used for late warnings of RRV outbreaks [13]. In other studies, a high abundance of several mosquito species (*Ae. vigilax*, *Cx. annulirostris*, *Anopheles annulipes*, *Cx. australicus*, *Cq. linealis*, *Ae. camptorhynchus*, and *Culex globocoxitus*) correlated with an increased number of RRV cases under most temporal lags [14,15,16]. However, under several other lags, this abundance was associated with a decrease in RRV case numbers. In a number of other models, no significant relationship between mosquito abundance and RRV cases was observed. Mosquito abundance or density has been widely used in RRV prediction.

In Queensland, areas of high population density are predominantly located in coastal regions [17], and these regions also represent high-risk areas for RRV [18]. The warm and humid climate of Queensland’s coastal cities is beneficial for the breeding of saltmarsh and tidal wetlands’ mosquitoes (e.g., *Ae. vigilax*) and peridomestic mosquitoes (e.g., *Ae. notoscriptus*) [5,19]. In this study, we focused on three coastal cities, one in a tropical area and two in subtropical areas and aimed to observe whether mosquito abundance data have predictive value for forecasting mosquito-borne diseases, such as RRV, in Queensland.

Alongside mosquitoes, other factors (e.g., climate, weather, geographic factors, and socio-economic indices) can also influence the RRV transmission cycle and were thus included in the prediction. Through using these predictors and developing cross-validated models, we aimed to: (1) predict RRV incidence in three cities of Queensland; and (2) evaluate the predictive value of mosquito abundance in prediction. The development of a predictive tool for RRV could enhance disease management and control, not only for RRV but also for other mosquito-borne diseases. Furthermore, assessing the utility of mosquito data in such models can confirm the importance of vector surveillance and provide information for effective mosquito control.

## 2. Materials and Methods

### 2.1. Study Design and Study Area

This study developed RRV incidence predictive models in three cities in Queensland using RRV notifications, mosquito abundance, and meteorological, geographical, and socio-economic predictors.

**Figure 1:**
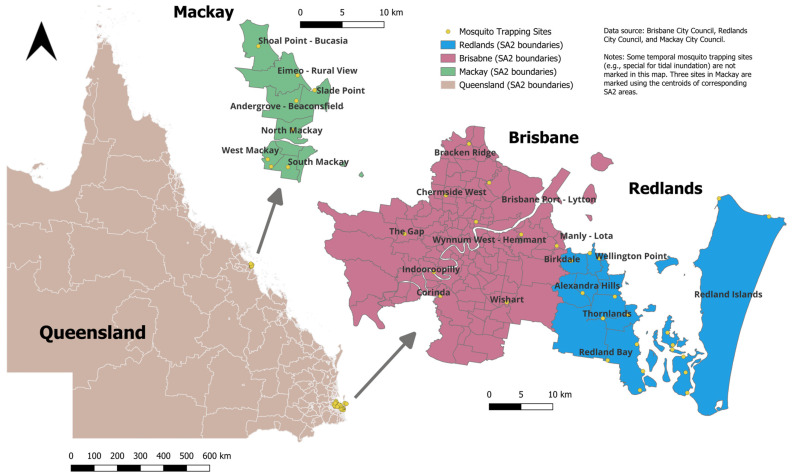
Research area (cities of Brisbane, Redlands, and Mackay) and the locations of the mosquito trapping sites. Redland Islands includes Lamb Island, North Stradbroke Island, Coochiemudlo Island, Karragarra Island, Russell Island, Macleay Island, and Peel Island.

Queensland is a large state with 528 Statistical Area Level 2 (SA2) areas, which is a functional geographical unit that generally has a population between 3000 and 25,000 people [20]. In this study, we included 7 SA2s from the city of Mackay, located on the central coast of Queensland, 10 SA2s from the city of Brisbane, and 8 from the city of Redlands, which are both located in South East Queensland near the coast (Figure 1). The city councils of these three cities have had ongoing vector surveillance programs for the past ten years; thus, they can provide sufficient data (more than 10 trapping sites in each city) for prediction. The main mosquito trapping sites are listed in the map except for several temporary sites in the city of Redlands [21]. These three cities mainly have a tropical or subtropical climate, with a hot, humid summer and a dry, warm winter (Table 1) [22]. Floods may happen in these cities in the summer, especially after heavy rain or thunderstorms. The diverse natural environment, which encompasses rainforests, wetlands, mangroves, and river catchments, presents mosquito breeding habitats. In addition to these natural environments, artificial containers situated around residential areas also provide suitable habitats for mosquito breeding [23]. The dominant mosquito species across coastal, urban, and hinterland areas can vary. The host species include birds and marsupials (such as possums, wallabies, and bandicoots) in natural habitats, and placental mammals (e.g., dogs, cats, and horses) in residential areas [2]. We conducted separate analyses in each city as they have varying meteorological, geographical, and socio-economic conditions, and the city councils may have different mosquito control and management plans. The research periods for Redlands, Brisbane, and Mackay were from January 2010 to June 2020, from July 2012 to December 2019, and from September 2007 to August 2020, respectively (Table 1).

### 2.2. Data Collection and Collation

Daily de-identified notifications of RRV via SA2 areas within the research period (Table 1) in the cities of Brisbane, Redlands, and Mackay were acquired from the Queensland Department of Health. Confirmation of a RRV case was defined as virus isolation, virus detection via nucleic acid testing, immunoglobulin G (IgG) seroconversion, or a significant increase in IgG antibody level [24]. A probable RRV case requires the detection of RRV immunoglobulin M (IgM) and IgG, except when IgG has also been detected more than three months previously. Both confirmed cases and probable cases were notified [24]. An annual human population estimate in each included SA2 area during the study period was obtained from the Australian Bureau of Statistics [25]. The weekly incidence rates were calculated by dividing the number of RRV cases by the total population of a given area in a week.

Daily weather data for the study periods were acquired from the Australian Bureau of Meteorology for rainfall, air temperature, relative humidity, evaporation, evapotranspiration, and vapour pressure [26]. Long-term climatic indices, such as La Niña events, El Niño episodes, and the SOI, during the study period were sourced from the Australian Bureau of Meteorology [26]. The socio-economic index for areas (SEIFA), which indicates the socio-economic advantages and disadvantages of an area, including education, occupation, and economic resources, was collected from the Australian Bureau of Statistics [25]. The monthly normalized difference vegetation index (NDVI) was obtained from the Bureau of Meteorology website. The week number of a year was also included as a predictor.

Total mosquito abundance was acquired from the city councils of Brisbane, Redlands, and Mackay [27,28,29]. The main mosquito trapping sites are listed in Figure 1. Trapping can be conducted for routine surveillance or for special purposes (e.g., after heavy rain or tidal inundation). Several temporary trapping sites in Redlands are not indicated in Figure 1, but the data from these sites were included in the analysis. In Brisbane and Redlands, the abundance of four mosquito species, *Cx. annulirostris*, *Ae. vigilax*, *Ae. notoscriptus*, and *Ae. vittiger*, which can be related to RRV incidence [30], were also included in the analyses. The mosquito species data were not available for Mackay. The weekly average abundance per trap was calculated for analysis.

Exposures were extracted and summarised as weekly data in SA2 areas. In this study, the years start from January and the weeks start from Monday. When aggregating daily data to weekly data, if not all the daily data in a week were available, the average value of non-missing data was calculated for the week. When estimating weekly data using annual or monthly data (e.g., NDVI), the data were converted to daily data via linear interpolation and were then summarised as weekly data by calculating an average. The spatial, meteorological, and geographical data collected in map format were first transformed and aggregated in the Quantum Geographic Information System (QGIS) 3.30.1 [31] and were then linked to the other data via the SA2s.

### 2.3. Predictor Selection

Detection of RRV in humans requires weeks, from infection to symptom development, and then to laboratory testing. This was modelled using lags of one or more weeks in predictors. Two lag times were selected for each predictor, considering lags of up to one year for the meteorological and geographical predictors (Table 1), and lags of up to 3 months for mosquito abundance. Lags for each predictor were selected according to the maximum positive and minimum negative cross-correlation function (CCF) values, which calculated the correlations between lagged variables and RRV cases. In this study, exposures were the potential impact factors of RRV transmission regardless of lags (e.g., mosquito abundance or rainfall). The exposures at specific lags, including lag zero, which represents the current value of an exposure, were predictors (e.g., rainfall at lag 1). In addition, a weighted moving average of recent RRV cases was generated as 4/10 of RRV cases at lag 1 plus 3/10 of RRV cases at lag 2 plus 2/10 of RRV cases at lag 3 plus 1/10 of RRV cases at lag 4. The recent RRV case variable was used as an autocorrelation term for predicting future RRV incidence.

In Redlands, a number of mosquito trapping sites are temporary; so, the lagged abundance in previous weeks was not available. Using lagged mosquito abundance predictors can cause many missing values and decrease the performance of the models; thus, the lagged mosquito abundance was not included in the analysis for Redlands. 

To obtain a parsimonious predictor set that had strong epidemiological and statistical correlations with RRV incidence, a five-step variable selection process based on the purposeful selection of covariates strategy proposed by Hosmer and Lemeshow [32] was developed (Supplementary Text S1). The current values of all predictors and their lags, together with the recent RRV case variable, were included in the variable selection process.

### 2.4. Model Building

A standard negative binomial generalised linear model demonstrably performs well with RRV data (Supplementary Text S1) [33]; thus, it was used for predicting RRV incidence in this analysis. A time-series cross-validation approach was applied to allow for the implementation of lagged predictors and to avoid using later data to predict earlier data. A long time period in cross-validation datasets was beneficial for analysing the effects of long-term climate exposures and seasonal variations. In contrast, to avoid possible effects of the revised case definitions on RRV prediction in 2013 and in 2016 (e.g., all validation datasets were defined by the new definitions), a shorter time for validation was preferred. Two to four years of data were selected in cross-validation as a trade-off. In Brisbane and Redlands, three training sets and validation sets were generated, while in Mackay, the research period was long (>10 years) and four counterparts were generated. 

The predictors were selected for each training set in each city. The Bayesian information criteria and mean absolute error were calculated but were more useful in comparing model performance rather than evaluating the performance of a single model. Measuring accuracy based on observed RRV cases (e.g., the predicted value lying between 90% and 110% of the real value is considered an accurate prediction) works well with RRV cases greater than twenty but is not appropriate when RRV cases were zero or one (as it often occurs). The predicted RRV trends were used to illustrate the performance of the models. The standardised coefficients were calculated to evaluate and compare the epidemiological effects of predictors on RRV incidence. Details of the methods are provided in Supplementary Text S1.

The trends of mosquito abundance and log-transformed RRV cases in three cities was plotted. The Spearman correlation of mosquito abundance and CCF values of RRV and mosquito abundance at weekly lags up to 3 months in the cities of Redlands and Brisbane were plotted. All analyses and figures were performed and produced with “Modern Applied Statistics with S (MASS)” [34], “pscl” [35] in R 4.1.0 (R Core Team, Vienna, Austria, 2021) [36], and QGIS 3.30.1 [31] using GCS_GDA_1994 Geographic Coordinate Systems. The R code of this study is provided in Supplementary Text S2. 

### 2.5. Ethics Statement

This study was approved by the University of Queensland Human Research Ethics Committee A (2019/HE002772).

## 3. Results

**Figure 2:**
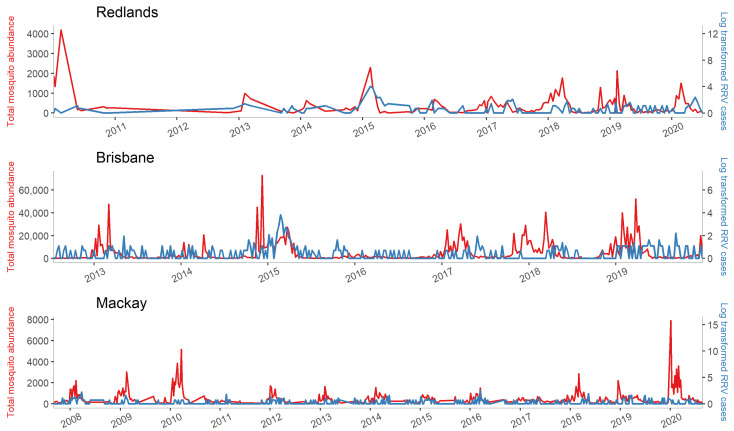
Weekly total mosquito abundance and log-transformed RRV cases in Redlands, Brisbane, and Mackay in QLD. The red line indicates the trend of total mosquito abundance, while the blue line denotes the trend of log-transformed RRV cases.

The trends of weekly total mosquito abundance and log-transformed RRV cases in the three cities are described in Figure 2. In Redlands, the seasonal variations were relatively clear for mosquito abundance but less so for RRV cases, and missing values in 2010, 2013, and 2015 caused the wide peaks (sharp and triangle-like shapes). In Brisbane, mosquito abundance had obvious seasonal variations; however, RRV notifications were reported throughout the year and were not strongly correlated with mosquito abundance. In Mackay, both mosquito abundance and RRV cases varied seasonally. The numbers of cases were comparatively low and were not strongly correlated with mosquito abundance. The trends of four mosquito species abundance are provided in Supplementary Figure S1. The species-specific abundances were also not correlated with the RRV cases. 

**Figure 3:**
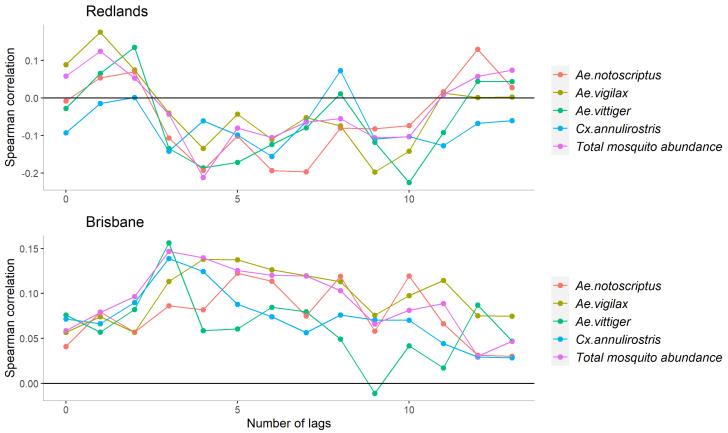
Cross−correlation function values of RRV and mosquito abundance at weekly lags up to 3 months in Redlands and Brisbane, QLD.

The CCF values displayed in Figure 3 show that the abundance of *Aedes* spp. mosquitoes in Redlands was positively correlated with RRV incidence at lags of 2–3 and 12–13 weeks and negatively correlated with RRV incidence under the other lags. *Cx. annulirostris* was negatively associated with disease incidence at all lags except for the lag of 8 weeks. However, the correlation plot (Supplementary Figure S2) indicates that all mosquito abundances were positively correlated with each other. In Brisbane, the abundance of all mosquito species and total abundance were positively associated with RRV incidence (weak correlations with Spearman correlation coefficients mainly between 0 and 0.15) under almost all lags.

In Redlands, our analysis demonstrated a negative correlation between RRV incidence and vapour pressure 8 months prior, as well as El Niño episodes one year ago (Table 2). Conversely, a positive association was found between RRV incidence and the recent RRV case variable. In Brisbane, RRV incidence was positively associated with recent RRV cases, vapour pressure two months prior, and the abundance of some mosquito species and was negatively associated with predictors including relative humidity and vapour pressure 7 to 8 months prior, and *Ae. vigilax* abundance one month prior (Table 2). In Mackay, the incidence rates of RRV had obvious seasonal variations, and exhibited a significant relationship with the week number of the year. Furthermore, a positive correlation with recent cases was observed. In contrast, negative associations were found between RRV incidence and weather predictors (rainfall and range of temperatures). The recent RRV cases variables were selected in all training sets, and mosquito-related predictors were only selected in two training sets for Brisbane and two for Mackay. Rainfall, vapour pressure, and relative humidity were frequently selected as important predictors (Supplementary Table S1). None of the socio-economic indices were selected as predictors.

**Figure 4:**
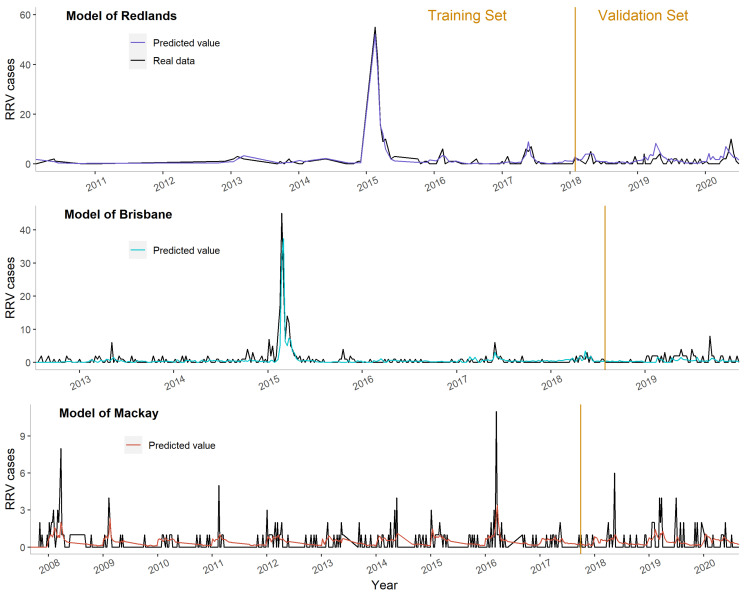
Predictive trends of RRV incidence in the cities of Redlands, Brisbane, and Mackay.

In Redlands, the numbers of observations were less than 1000, and the predictors selected for model building were less conserved across the different training sets. In Brisbane and Mackay, when the numbers of observations were above 1000, the predictors selected were largely the same (more stable). The predictive models worked well in all three sites and performed slightly better in the training sets than in the validation sets (Figure 4). The models predicted the peaks well in the cities of Redlands and Brisbane. However, in Mackay, the peaks in RRV cases were at low numbers (around 5–10 cases per week) and was thus hard to predict. 

## 4. Discussion 

We predicted RRV incidence in three cities (Brisbane, Redlands, and Mackay) of Queensland, Australia, using the recent RRV case variable, mosquito abundance, and meteorological, geographical, and socio-economic predictors with selected lags. The model-building process (including the predictor selection strategy) performed well, even under small geographical areas. Around 10 years of good-quality mosquito data in each city provided us with the opportunity to evaluate the utility of mosquito abundance in RRV prediction. Mosquito abundance was generally valuable for prediction in Brisbane but not in the other two cities. 

In Brisbane, RRV cases can occur under all seasons, but mosquitoes mainly breed from the summer to early autumn. Increased abundance of all mosquito species is associated with higher numbers of RRV cases in the following three months based on positive CCF values in the bivariate analysis. In the multivariate models, increasing abundance of *Ae. vigilax* at one month prior and of *Cx. annulirostris* at three months prior were related to a decreased RRV incidence, while the increased current abundance of *Cx. annulirostris* was related to a higher incidence. One possible explanation for this is that the activity and behaviour of mosquitoes, and the effects of mosquito abundance on RRV dynamics, were affected by the weather predictors. Weather can be a confounder, which will not influence the final predictive results but will influence the directions or values of the predictor coefficients, either the mosquito abundance or weather. We aimed to use mosquito abundance data for our predictions rather than to detect or control the confounding effects; hence, confounding was not a primary problem in this study. Our study demonstrated that mosquito abundance data have predictive value in forecasting RRV incidence in Brisbane.

None of the mosquito abundance variables were important predictors in the Redlands model. There are very few residents but many visitors in the islands of Redlands, such as North Stradbroke Island, Macleay Island, and Russell Island. Visitors may have left Redlands after getting infected with RRV and then be notified in other cities. This would produce underenumeration for Redlands and biased associations toward the null. We have no ability to correct this bias using our data. However, the predictive curve matched the real RRV notification trends in Redlands quite closely. Disease incidence was found to be spatially related to the mosquito biting exposure [37], which is much more useful than the mosquito abundance as a predictor. 

The occurrence of RRV was sporadic from the late spring to early autumn in Mackay, with the mosquito abundance rising under similar seasons. However, the number of RRV cases was comparatively low each week no matter whether the mosquito abundance was high or not. Mosquito abundance had a weak impact on RRV transmission but was not as strongly associated as the seasonal variations, recent cases, and weather predictors (e.g., rainfall and range of temperature). 

The predominant mosquito species in both Brisbane and Redlands was *Ae. vigilax*, which is a saltwater mosquito with habitats in the coastal wetlands or in the mangrove forests [38]. In Brisbane, *Cx. annulirostris*, which breeds in freshwater (e.g., freshwater wetlands, pools, and containers) and is active throughout the year, is also a commonly trapped mosquito species [39]. *Cx. annulirostris* has been proven to be susceptible to Barmah Forest virus infection (another leading *Alphavirus* infection in Australia) and Japanese encephalitis, but is relatively inefficient [40]. *Ae. notoscriptus* is another competent RRV vector species that is widespread in Australia [41,42], but was not a dominant and important species in our study area.

Our analysis indicated that the recent RRV case variable was an important predictor across all cities. Additionally, a number of meteorological variables, such as rainfall, relative humidity, and vapour pressure, were selected for prediction. These variables can influence mosquito breeding, larval survival, and overall mosquito activity, and thus exhibit substantial impacts on RRV transmission dynamics [33]. A possible explanation that socio-economic predictors were not included in modelling was that the research was conducted in three cities, where the socio-economic conditions were basically the same. 

The predictors selected in the training sets became stable when the numbers of observations were above 1000. In Mackay, the numbers of RRV cases were relatively low without obvious outbreaks in a research period spanning 13 years, and this led to difficulties in its prediction. In Redlands, the lagged mosquito data were not included in our analyses as they caused considerable missing values (512 missing values in 700 observations), thus negatively impacting its model’s performance. Sufficient RRV notifications are required to retrieve a reliable predictor set and good predictive performance.

Vector surveillance can not only provide data for predicting disease trends but also indicate sites or areas with high vector abundance and provide suggestions for conducting mosquito control programs. In addition to weekly mosquito trapping, all three city councils perform mosquito treatments routinely and after heavy rain, floods, or tidal inundation. The treatment aims for mosquito control rather than surveillance or detection of the arboviral diseases; therefore, these data are seldom utilised in predictions. Previous studies have found that that mosquito control (especially freshwater mosquito control) is related to lower RRV notifications in Queensland [43,44], but this parameter is not included in the analyses.

The reservoir hosts, a key element in the RRV transmission cycle, were not included in this study due to a lack of host population distribution data in the relevant geographical areas. The population and distribution of the reservoir hosts, such as koalas [45], and their RRV exposure can be valuable in the context of disease prediction. At present, data available for analysing the mosquito–host associations are also limited. 

Routine surveillance of several flood- or tidal-influenced mosquito trapping sites could provide continuous data to calculate lagged mosquito abundances for predicting infectious diseases. The abundances of *Ae. vigilax*, *Cx. annulirostris*, and *Ae. notoscriptus* were valuable for predicting RRV incidence in Brisbane, the largest city in Queensland. Isolation of the virus in mosquitoes would be helpful for its prediction; however, this will be complex and costly [11]. Mosquito trapping is an important method for the surveillance of mosquito diversity and, ultimately, mosquito-borne diseases.

## 5. Conclusions

By utilising data on mosquito abundance, recent RRV cases, and meteorological, geographical, and socio-economic data, we were able to effectively forecast RRV incidence in three cities of Queensland. Our findings revealed that RRV transmission patterns significantly vary across cities. Mosquito abundance can be used to predict RRV infection in several areas. Mosquito abundance, vapour pressure, and relative humidity were valuable for prediction in Brisbane, while seasonal variation dominates the trends of disease incidence in Mackay. In Redlands, the data may not have been sufficient to generate a stable predictor set. However, the model provided satisfactory predictive results. The recent RRV case variable was a strong and valuable predictor in general. The availability of more detailed mosquito data (with RRV isolations) could improve the accuracy in prediction. Mosquito surveillance not only plays pivotal roles in forecasting RRV activity, but also in the management and control of all mosquito-borne diseases. This study adds to the evidence that mosquito abundance data can be used in predicting RRV incidence and demonstrates that diverse factors dominate RRV transmission in cities with varying environmental characteristics in Queensland. The analysis demonstrates its use in predicting incidence rates of RRV (and other mosquito-borne disease) in small geographical areas. Future studies with broader ecological data exploring the relationships between vectors and non-human reservoir hosts could provide valuable insights into the complex associations within RRV ecology and thus aid in the precise predictions and early warnings of RRV.

## Figures and Tables

**Table 1:** Description of research data. SA2 = Statistical Area Level 2; RRV = Ross River virus.

	Redlands	Brisbane	Mackay
Climate of the research area	Subtropical climate with hot, humid summers, and dry, moderately warm winters. Thunderstorms occasionally occur during the summer and cause waterlogging.		Tropical climate with hot, humid summers, and dry, warm, sunny winters with cool nights. Tropical cyclones, thunderstorms, and subsequent floods occasionally occur during the summer.
Geographical situation of the research area	Coastal city with diverse natural environment, including several islands, coastline, bushlands, national parks, and waterways. Elevation: from 0 m to 233 m.	Subcoastal city with substantial residential and agricultural lands and natural environments, including bushlands, forests, mountains, and the Brisbane River. Elevation: from 3 m to 512 m.	Coastal city at the mouth of the Pioneer River with both residential areas and natural environments, including a long coastline and rainforests, islands, and gardens. Elevation: from 0 m to 65 m.
Socio-economic situation of the research area	The mainland of Redlands is made up of urban and suburban areas close to Brisbane City. Islands including North Stradbroke Island have few residents but have common visitors. Average population density of around 307 persons per square km.	Brisbane is the capital of Queensland and Australia’s third-largest city. Populous urban city (average population density: around 1270 persons per square km).	Mackay is a moderate city with an average population density around 595 persons per square km, and is popular with visitors; it is also known as the “sugar capital” of Australia.
Research period	1 January 2010–30 June 2020	1 July 2012–30 December 2019	1 September 2007–30 August 2020
Number of SA2 areas included in this analysis	8	10	7
Observations with mosquito abundance data (total RRV cases)	1231 (278)	3495 (376)	3349 (246)
Mosquito data (weekly data at SA2s)	Abundance of *Cx.annulirostris*, *Ae.vittiger*, *Ae.notoscriptus*, *Ae.vigilax*, and total mosquitoes and two of the weekly lags of these abundances within three months.		Total mosquito abundance and two weekly lags of total abundance within three months.
Meteorological, geographical, and socio-economic exposures (weekly data at SA2s) and recent RRV cases	Max air temperature, range of air temperature, relative humidity at the time of maximum temperature, relative humidity at the time of minimum temperature, rainfall, vapour pressure at 3 p.m., pan evaporation, evapotranspiration, Southern Oscillation Index, El Niño events, La Niña events, normalized difference vegetation index, and two weekly lags of these exposures within one year. Socio-economic index for areas, week number of a year, and the log-transformed value of weighted moving average of recent RRV cases in the past month.		

**Table 2:** Predictors selected for each city and the standardised coefficients (sorted via absolute value of standardised coefficients). ^†^ Redlands and Brisbane data generated the third training set and Mackay the fourth training set. ^††^ Recent cases = log (weighted moving average of recent RRV cases in the past month + 1); Week = week number in a year; TempR = range of air temperature; RHMin = relative humidity at the time of minimum temperature; RHMax = relative humidity at the time of maximum temperature; and NDVI = normalized difference vegetation index. All the lags are weekly lags. ***: *p*-value < 0.001, and *: *p*-value < 0.05.

Dataset ^†^	Predictors ^††^	Standardised Coefficients
Redlands	Vapour pressure lag 35	−0.630 ***
	El Niño lag 51	−0.515 ***
	Recent cases	0.515 ***
	RHMin	0.224
	RHMax lag 7	0.159
	Rainfall	−0.018
Brisbane	Recent cases	0.463 ***
	RHMin lag 36	−0.332 ***
	Vapour pressure lag 9	0.222
	Vapour pressure lag 29	−0.196
	*Ae.vigilax* lag 5	−0.156
	Week	−0.131
	*Cx.annulirostris* lag 14	−0.126
	*Cx.annulirostris*	0.119 *
	Evapotranspiration lag 12	−0.105
	RHMax	0.088
	*Ae.notoscriptus* lag 14	0.070
	Vapour pressure	−0.030
	*Cx.annulirostris* lag 4	0.024
	Total mosquito abundance lag 13	0.018
	*Ae.notoscriptus* lag 6	−0.008
	*Ae.vigilax* lag 1	−0.007
	Total mosquito abundance lag 4	−0.006
Mackay	Week	−0.520 ***
	Recent cases	0.293 ***
	Rainfall lag 45	−0.231
	TempR lag 51	−0.056
	NDVI lag 49	0.009

## Data Availability

The data that support the findings of this study are available from the Queensland Department of Health upon reasonable request.

## Supplementary Materials

The following supporting information can be downloaded at: https://www.mdpi.com/article/10.3390/biology12111429/s1, Supplementary Text S1. Details of methods. Supplementary Text S2. R code of prediction of Ross River virus incidence using mosquito data in three cities of Queensland, Australia. Supplementary Figure S1. Weekly abundance of four mosquito species in Brisbane and Redlands, QLD. Supplementary Figure S2. The Spearman correlation of abundance of all mosquitoes and four mosquito species in Redlands and Brisbane, QLD. Supplementary Table S1. Predictors selected in each training set of the modelling process and the standardised coefficients (sorted by absolute value of standardised coefficients). References [46,47,48] are cited in the supplementary materials.
