# Supplementary material for: Prediction of Ross River Virus Incidence Using Mosquito Data in Three Cities of Queensland, Australia
Source: Biology (Basel). 2023 Nov 13;12(11):1429. doi: 10.3390/biology12111429 (PMC10669834; doi:10.3390/biology12111429)
Supplement: Supplementary file 1 [file biology-12-01429-s001.zip › Supplementary Table_S1_Predictors selected.pdf]

Supplementary Material

Table S1. Predictors selected in each training set of the modelling process and the standardised coefficients (sorted by absolute value of standardised coefficients).

†Redlands and Brisbane data generated three training and validation sets, and Mackay generated four counterparts. ††SC = Standardised Coefficients; Recent cases = log (weighted moving average of recent RRV cases in the past month + 1); Week = Week number in a year; TempMax = Maximum air temperature; TempR = Range of air temperature; RHMin = Relative humidity at the time of minimum temperature; RHMax = Relative humidity at the time of maximum temperature; SOI = Southern Oscillation Index; IEO = Index of Education and Occupation; IRSAD = The Index of Relative Socio-economic Advantage and Disadvantage; NDVI = Normalized Difference Vegetation Index. All the lags are weekly lags. ***p value < 0.001, *p value < 0.05.

**Dataset†**	**Redlands**	**Brisbane**	**Mackay**
	Predictors, SC††	Predictors, SC††	Predictors, SC††
Training set 4 (Obs.)	-	-	2007.9.1-2017.9.30 (2417)
Validation set 4 (Obs.)	-	-	2017.10.1-2020.8.30 (932)
	-	-	Week, **-0.520*****<br>Recent cases, **0.293*****<br>Rainfall lag 45, -0.231<br>TempR lag 51, -0.056<br>NDVI lag 49, 0.009
Training set 3 (Obs.)	2010.1.1-2018.1.30 (700)	2012.7.1-2018.7.30 (2761)	2007.9.1-2015.9.30 (1894)
Validation set 3 (Obs.)	2018.2.1-2020.6.30 (531)	2018.8.1-2019.12.30 (734)	2015.10.1-2017.10.30 (571)
	Vapour pressure lag 35, **-0.630*****<br>El Niño lag 51, **-0.515*****<br>Recent cases, **0.515*****<br>RHMin, 0.224<br>RHMax lag 7, 0.159<br>Rainfall, -0.018	Recent cases, **0.463*****<br>RHMin lag 36, **-0.332*****<br>Vapour pressure lag 9, 0.222<br>Vapour pressure lag 29, -0.196<br>*Ae.vigilax* lag 5, -0.156<br>Week, -0.131<br>*Cx.annulirostris* lag 14, -0.126<br>*Cx.annulirostris*, **0.119***<br>Evapotranspiration lag 12, -0.105<br>RHMax, 0.088<br>*Ae.notoscriptus* lag 14, 0.070<br>Vapour pressure, -0.030<br>*Cx.annulirostris* lag 4, 0.024<br>Total mosquito abundance lag 13, 0.018<br>*Ae.notoscriptus* lag 6, -0.008	Week, **-0.513*****<br>Rainfall lag 45, **-0.339***<br>Recent cases, **0.249*****<br>El Niño lag 46, -0.144<br>TempR lag 51, -0.101<br>NDVI lag 49, -0.026

		Ae.vigilax lag 1, -0.007	
		Total mosquito abundance lag 4, -0.006	
Training set 2 (Obs.)	2010.1.1-2016.1.30 (338)	2012.7.1-2016.7.30 (1733)	2007.9.1-2013.9.30 (1431)
Validation set 2 (Obs.)	2016.2.1-2018.2.30 (370)	2016.8.1-2018.8.30 (1028)	2013.10.1-2015.10.30 (487)
	Recent cases, **0.705*****	El Niño lag 23, **-0.647*****	Rainfall lag 45, -0.354
	Vapour pressure lag 35, **-0.438***	TempR, -0.447	Week, -0.336
	IRSAD, **0.351*****	*Ae.vigilax* lag 5, -0.410	Recent cases, **0.303*****
	Evapotranspiration lag 10, 0.303	Recent cases, **0.376*****	Evapotranspiration lag 30, -0.279
	TempMax lag 10, -0.123	Pan evaporation lag 12, **-0.357***	El Niño lag 46, -0.189
	Week, -0.062	*Cx.annulirostris* lag 14, -0.332	Vapour pressure, -0.158
	TempR lag 9, -0.058	Week, -0.244	Total mosquito abundance, **0.155***
	Pan evaporation lag 16, 0.012	Vapour pressure lag 9, 0.224	RHMax, 0.111
		RHMax, -0.160	TempR lag 51, -0.058
		Ae.vigilax lag 1, -0.134	Vapour pressure lag 4, 0.036
		Cx.annulirostris, **0.119***	Vapour pressure lag 27, -0.006
		Ae.vittiger lag 4, 0.109	
		Vapour pressure lag 29, -0.105	
		Ae.notoscriptus lag 14, 0.099	
		Cx.annulirostris lag 4, 0.092	
		TempMax lag 11, 0.045	
		Total mosquito abundance lag 13, 0.033	
		Ae.notoscriptus lag 6, -0.027	
		Total mosquito abundance lag 4, -0.014	
		Ae.vittiger lag 10, 0.001	
Training set 1 (Obs.)	2010.1.1-2014.1.30 (152)	2012.7.1-2014.7.30 (824)	2007.9.1-2011.9.30 (963)
Validation set 1 (Obs.)	2014.2.1-2016.2.30 (201)	2014.8.1-2016.8.30 (909)	2011.10.1-2013.10.30 (498)
	IEO, 0.675	SOI, **0.319***	Week, -0.416
	NDVI lag 47, 0.342	NDVI, -0.270	Recent cases, **0.377*****
	SOI lag 25, -0.285	RHMin lag 25, -0.249	NDVI lag 49, 0.248
	Rainfall, -0.263	Vapour pressure lag 29, -0.214	Vapour pressure, 0.217
	Pan evaporation lag 16, 0.120	Rainfall lag 10, 0.132	Vapour pressure lag 27, -0.212
	RHMin lag 24, 0.117	SOI lag 42, 0.109	El Niño lag 46, -0.152
	Rainfall lag 2, 0.104	Recent cases, 0.067	Vapour pressure lag 4, -0.125
	Recent cases, 0.077	TempR lag 29, 0.046	Total mosquito abundance, 0.106
	IRSAD, 0.030		TempR lag 51, -0.085
