# Supplementary material for: Prediction of Ross River Virus Incidence Using Mosquito Data in Three Cities of Queensland, Australia
Source: Biology (Basel). 2023 Nov 13;12(11):1429. doi: 10.3390/biology12111429 (PMC10669834; doi:10.3390/biology12111429)
Supplement: Supplementary file 1 [file biology-12-01429-s001.zip › Supplementary Text_S1_Details of methods .pdf]

Supplementary Material

Text S1. Details of methods

Model building

Predictor definition

Two time lags were selected for predictors, allowing lags of up to one year for some weather and climate predictors and up to 3 months for mosquito abundance using the maximum positive and minimum negative of the cross-correlation function value, which calculates the correlation between lagged predictors and Ross River virus (RRV) cases. Due to the time required for mosquito breeding, transmission to reservoir hosts, the incubation period in humans, and lags in case reporting, we considered that lags less than one year could have ecological meaning for associations between weather and climate predictors and RRV incidence. Longer lags may plausibly indicate causal links between climate and vegetation cover, or, vegetation cover and host populations, which then influence RRV incidence (Ng et al., 2014), but it was beyond the scope of this study to investigate these longer lags.

The weighted moving average of RRV recent cases was generated by 4/10 of RRV cases at lag 1 (in one previous week), plus 3/10 of RRV cases at lag 2, plus 2/10 of RRV cases at lag 3 plus 1/10 of RRV cases at lag 4. This calculation assumed that recent RRV cases might have an arithmetic decaying correlation with current cases as the order of the lags increases. Log transformed values of the moving average were applied in models, in line with the nature of log-linear models for the analysis of count outcomes. One was added to moving averages before log transforming to adjust for zero values in data of RRV notifications (MaCurdy & Pencavel, 1986).

Predictor selection process

Predictors with p values greater than 0.1 or standardised regression coefficients less than 0.1 were excluded based on the univariate analysis as having weak or no impact on RRV incidence. The Spearman correlation between each two predictors was calculated. Predictors with Spearman correlations greater than 0.9 were excluded for reasons of redundancy and possibility of violating the hypothesis of the models. Predictors having higher Spearman correlation values (>0.95) or having high correlations with more predictors were excluded first, then predictors were excluded with the consideration of retaining as many predictors as we can in the analysis.

Then, a repeated backward stepwise screen and reassessment process was used to screen predictors improving model fit of predictive models. Predictors with Variance Inflation Factor (VIF) greater than 5 were removed at the start of each iteration. The VIF was calculated based on inflation of coefficient variance in models and used to address the magnitude of multicollinearity. The predictor with lowest Bayesian Information Criterion (BIC) increment was

removed until the total BIC increment reached 0.5%. The removed predictors were then considered in the model one at a time, and those with VIF less than 10 and BIC decrement greater than 0.1% were included and the next iteration commenced. We continued this process until no predictor was excluded or included, two consecutive iterations returned the same variable set, or it reached 11 iterations. This process was performed in the meteorological predictor set and the geographical and socio-economic predictor set separately, then in all predictors selected from the two sets together with mosquito abundance and recent RRV cases.

Finally, to avoid predictor collinearity, predictors with VIF greater than five were excluded. The remaining predictors were selected for model building. Further details of this process are shown in Figure T1.

Predictors were selected with considerations of statistical significance, standardised regression coefficients, multicollinearity, overall fit and correlation among predictors. The process based on these multiple criteria is likely to identify the most important predictors with appropriate lags for prediction.

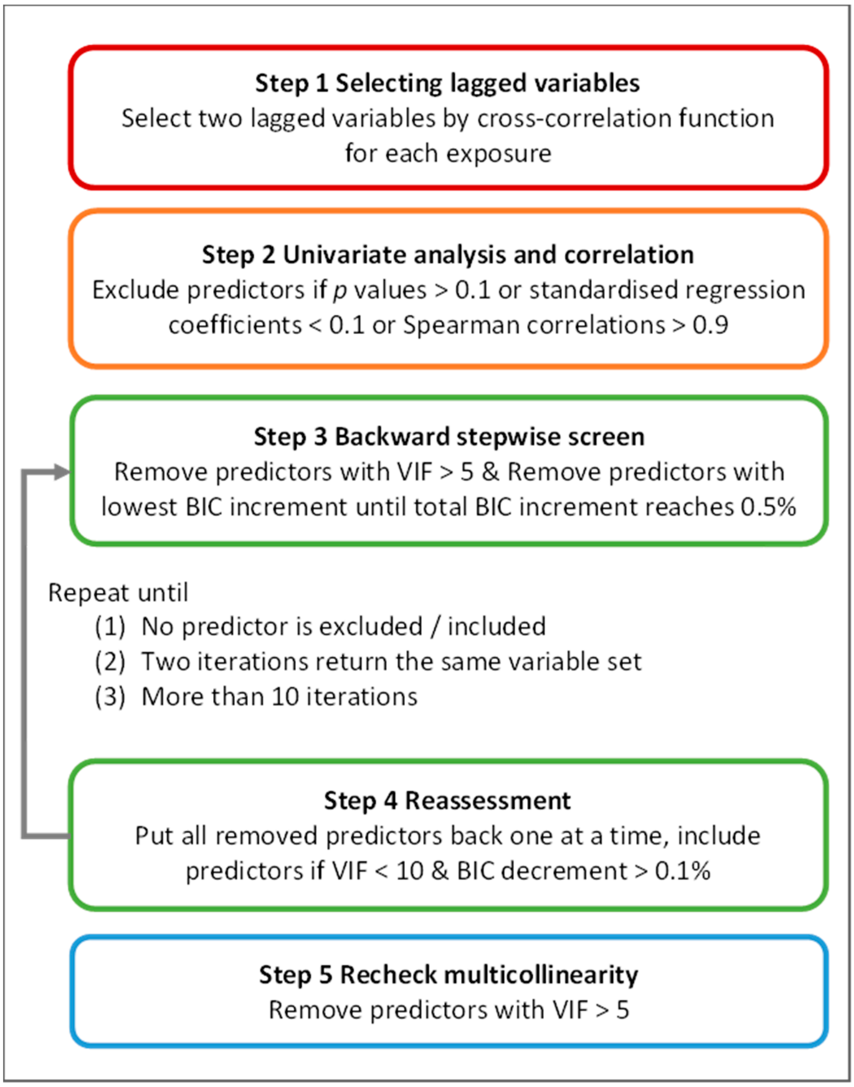

Figure T1. Predictor selection flowchart.

Cross-validation

As the longitudinal data used for modelling included lagged effects of predictors, a time-series cross-validation approach was applied to generate contiguous blocks of data and to avoid using later data to predict earlier data. In Brisbane and Redlands, three training sets and validation sets were generated, while in Mackay, four counterparts were generated (Table 2 in the main text). For each training dataset, predictors (weather, climate, geographical predictors, socio-economic indices, mosquito abundance at appropriate lags, and the moving averages of recent RRV cases) were selected for building models. To ensure that population size differences did not inflate or diminish effect sizes, we offset the population size at SA2 level in our count models.

Modelling method

Generalised linear models are widely applied in predicting Ross River virus (RRV) notifications, incidence rates and outbreaks (Qian et al., 2020). Linear models are simple and straightforward in explaining the relationship between predictors and RRV infection outcome variables. As RRV data are at relatively small spatial and temporal resolution (weekly data at SA2 areas), the weekly RRV notifications generated were rare counts with excess zeros. Data at a lower spatial or temporal resolution (e.g., monthly data) have fewer zeros but may lose information, especially for daily weather data. The negative binomial distribution could account for the effect of over-dispersion of the data possibly caused by excess zeros. We assessed performance of generalised linear models, zero-inflated models, and non-linear models for prediction in our previous work. The standardised negative binomial generalised linear model was demonstrated to be the most appropriate method for modelling (Qian et al., 2022).

A generalised linear model consists of a random component, a linear predictor and a smooth and invertible linearising link function (Fox, 2015),

$$g(E(Y_i)) = \alpha + \beta_1 X_{i1} + \beta_2 X_{i2} + \cdots + \beta_K X_{iK} \tag{1}$$

where $g(\bullet)$ is the link function, $E(Y_i)$ is the expectation of the response variable for the *i*th observation, X_{iK} is the variable and *K* is the number of variable, α is the intercept, β_K is the coefficient of the *K*th variable.

Model performance

The evaluation of the models is mainly based on the predictive trends of RRV.

References

Fox, J. (2015). *Applied regression analysis and generalized linear models*. Sage Publications.

MaCurdy, T. E., & Pencavel, J. H. (1986). Testing between competing models of wage and employment determination in unionized markets. *Journal of Political Economy*, *94*(3, Part 2), S3-S39.

Ng, V., Dear, K., Harley, D., & McMichael, A. (2014). Analysis and prediction of ross river virus transmission in New South Wales, Australia [Review]. *Vector-Borne and Zoonotic Diseases*, *14*(6), 422-438. https://doi.org/10.1089/vbz.2012.1284

Qian, W., Harley, D., Glass, K., Viennet, E., & Hurst, C. (2022). Prediction of Ross River virus incidence in Queensland, Australia - Building and comparing models. *PEERJ*, *10*, 1-22. https://doi.org/https://doi.org/10.7717/peerj.14213

Qian, W., Viennet, E., Glass, K., & Harley, D. (2020). Epidemiological models for predicting Ross River virus in Australia: A systematic review. *PLoS Neglected Tropical Diseases*, *14*(9), e0008621. https://www.ncbi.nlm.nih.gov/pmc/articles/PMC7537878/pdf/pntd.0008621.pdf
