# Supplementary material for: Prediction of Ross River Virus Incidence Using Mosquito Data in Three Cities of Queensland, Australia
Source: Biology (Basel). 2023 Nov 13;12(11):1429. doi: 10.3390/biology12111429 (PMC10669834; doi:10.3390/biology12111429)
Supplement: Supplementary file 1 [file biology-12-01429-s001.zip › Supplementary Text_S2_R code.pdf]

Supplementary Material

Text S2. R code. Prediction of Ross River virus incidence using mosquito data in three cities of Queensland, Australia.

```
# R packages

library(reshape2)

library(openxlsx)

library(dplyr)

library(data.table)

library(raster)

library(ncdf4)

library(lubridate)

library(imputeTS)

library(tidyverse)

library(Hmisc)

library(corrplot)

library(ggplot2)

library(ggcorrplot)

library(caret)

library(MASS)

library(Metrics)

library(modelr)

library(pscl)
```

```
library(mgcv)

library(performance)

library(patchwork)
```

Data collation

```
# Function of calculating week numbers.

WeekNo <- function(myDate) {

  WeekNo <- ifelse(weekdays(as.Date(paste(format(as.Date(myDate),"%Y"), "01-01", sep = "-")))
== "Monday",

    as.numeric(format(as.Date(myDate), "%W")),

    as.numeric(format(as.Date(myDate), "%W")) + 1)

  return(WeekNo)

}


# Import the excel with the SA2 areas and corresponding grid numbers.

# This requires a spatial join of Queensland SA2 map and grid maps in ArcGIS.

GSA2 <- read.xlsx("C:/Users/qianwei/Desktop/Grid_SA2.xlsx", sheet = "Grid_SA2")


# Function of reading NC files and extracting climate data.

# Ipath is the import file path, and Epath is the export file path, Exva means the exposure
variable.

ExvaNC <- function(y) {

  Exva <- brick(paste(y, Ipath, sep = ""))
```

```
  dim(Exva)
  # Read the layers in the brick as a data frame
  # The number of layers is the third dimension of the original database
  Exva.df <- as.data.frame(Exva[[1:dim(Exva)[3]]], xy = T)
  Exva.df <- Exva.df %>%
    rename(Latitude = y, Longitude = x)
  Exva.j <- left_join(GSA2, Exva.df, by = c("Latitude", "Longitude"))
  Exva.m <- reshape2::melt(Exva.j, id.vars = c("SA2"), measure.vars = c(4:(dim(Exva)[3] + 3)))
  Exva.mm <- Exva.m[! (is.na(Exva.m$value)), ]
  Exva.s <- Exva.mm %>%
    group_by(variable, SA2) %>%
    summarise(Exvaavg = mean(value), Exvamax = max(value), Exvamin = min(value), n = n())
  write.xlsx(Exva.s, paste(Epath, y, ".xlsx", sep = ""))
}
```

Set the Ipath and Epath, then extract weather data from 1999 to 2020 to excel documents (use rainfall data as an example).

```
setwd("C:/Users/Qianwei/Downloads/")
Ipath <- ".daily_rain.nc"
Epath <- "C:/Users/Qianwei/Desktop/Rain"
for (y in 1999:2020) {
  ExvaNC(y)
```

```
}

# Aggregate the excel files extracted.
path <- "C:/Users/Qianwei/Desktop/"
filenames_list <- list.files(path = path, pattern = "\\.xlsx$", full.names = T)
All <- lapply(filenames_list, function(filename) {
  filename <- read.xlsx(filename, sheet = 1)
  filename
})

# Collate the aggregated data (use rainfall data as an example).
Rain <- do.call(rbind.data.frame, All)
tail(Rain)
Rain$Year <- as.numeric(substr(Rain$variable, 2, 5))
Rain$Month <- as.numeric(substr(Rain$variable, 7, 9))
Rain$Day <- as.numeric(substr(Rain$variable, 10, 12))
Rain$Date <- as.Date(with(Rain, paste(Year, Month, Day, sep = "-")), "%Y-%m-%d")
Rain$Week <- WeekNo(Rain$Date)
tail(Rain)
Rain.g <- Rain %>%
  group_by(SA2, Year, Week)
Rain.s <- Rain.g %>%
```

```
  summarise(Rainavg = mean(Exvaavg), Rainmax = max(Exvamax), Rainmin = min(Exvamin), 
Rainsd = sd(Exvaavg), n = sum(n))
head(Rain.s)
length(unique(Rain.s$SA2))
write.xlsx(Rain.s, "C:/Users/Qianwei/Desktop/Rain.xlsx")

# Collating data (other than NC files)
# NDVI
path <- "C:/Users/Qianwei/Desktop/NDVI"
filenames_list <- list.files(path = path, full.names = TRUE)
All <- lapply(filenames_list, function(filename) {
  data <- read.table(filename, header = FALSE, sep = " ", dec = ".", skip = 6, nrow = 681)
  data <- data[ , 2:842]
  data <- reshape2::melt(data, measure.vars = c(1:841))
  data$value[data$value == 99999.9] <- NA
  data <- data[!is.na(data$value), ]
  data$colno <- seq(length = nrow(data))
  data <- data[ , 2:3]
  data <- left_join(GSA2, data, by = c("colno"))
  NDVI <- data %>%
    group_by(SA2) %>%
    summarise(NDVIavg = mean(value, na.rm = TRUE))
```

```
  NDVI$Date <- as.numeric(substring(filename, 59, 64))
  NDVI
})
NDVI <- do.call(rbind.data.frame, All)


#Do interpolation
SA2 <- unique(NDVI$SA2)
NDVI.i <- data.frame()
for (i in 1:length(SA2)) {
  for (j in 1:12) {
  snum <- as.numeric(SA2[i])
  NDVI.sub <- dplyr::filter(NDVI.r, SA2 == snum & Month == j)
  NDVI.sub <- NDVI.sub %>%
    na_interpolation(option = "linear")
  NDVI.i <- dplyr::bind_rows(NDVI.i, NDVI.sub)
  print(paste(i, j, NDVI.sub[1, 5], sep = ","))
  }
}


#  Southern Oscillation Index
SOI <- read.xlsx("SOI.xlsx", sheet = 1)
Date <- read.xlsx("Date.xlsx", sheet = 1)
```

```
SOI.j <- left_join(SOI, Date, by = c("Year", "Month"))

SOI.g <- SOI.j %>%

  group_by(Year, Week)

SOI.s <- SOI.g %>%

  summarise(SOIavg = mean(Southern.Oscillation.Index), n = n())


# El Niño & La Niña

ELLA <- read.xlsx("ELLA.xlsx", sheet = 1)

Date <- read.xlsx("Date.xlsx", sheet = 1)

ELLA.j <- left_join(ELLA, Date, by = c("Year", "Month"))

ELLA.g <- ELLA.j %>%

  group_by(Year, Week)

ELLA.s <- ELLA.g %>%

  summarise(ELmedian = median(ElNino), LAmedian = median(LaNina), n = n())


# Human population.

setwd("C:/Users/Qianwei/Desktop/")

HP <- read.xlsx("HP.xlsx", sheet = "hp")

HP.m = reshape2::melt(HP, id.vars = c("SA2"), measure.vars = c(2:21))

HP.m$Year <- as.numeric(as.character(HP.m$variable))

# Set the date of the population as 1st July of each year

HP.m$Date <- as.Date(with(HP.m, paste(Year, 7, 1, sep = "-")), "%Y-%m-%d")
```

```
Date <- read.xlsx("C:/Users/Qianwei/Desktop/Date.xlsx", sheet = 2)

Datehp <- Date[c(1, 5)]

Datehp$Date <- as.Date(Datehp$Date, origin = "1899-12-30", format = "%Y-%m-%d")

HP.m <- HP.m[c(1, 3, 5)]


SA2 <- unique(HP.m$SA2)

HP.f <- data.frame()

for (i in 1:length(SA2)) {

  snum <- as.numeric(SA2[i])

  HP.sub <- dplyr::filter(HP.m, SA2 == snum)

  HP.j <- left_join(Datehp, HP.sub, by = c("Date"))

  HP.i <- HP.j %>%

    na_interpolation(option = "linear")

  HP.f <- dplyr::bind_rows(HP.f, HP.i)

  print(paste(i, HP.sub[1, 3], sep = ","))

}

HP <- HP.f


# Ross River virus data.

RRV <- read.xlsx("C:/Users/Qianwei/Desktop/RRV.xlsx", sheet = "RRV")

RRV.s <- RRV %>%

  group_by(Date, SA2) %>%
```

```
  summarise(RRVn = n())

RRV.j <- full_join(Date, RRV.s, by = c("Date"))

RRV.j$Date <- as.Date(RRV.j$Date, origin = "1899-12-30", format = "%Y-%m-%d")

RRV.c <- left_join(HP.f, RRV.j, by = c("Date", "SA2", "Week"))

RRV.c <- RRV.c[c(1:4, 8)]

RRV.c$RRVn[is.na(RRV.c$RRVn)] <- 0

SA2 <- unique(RRV.c$SA2)

RRV.f <- data.frame()

for (i in 1:length(SA2)) {

  snum <- as.numeric(SA2[i])

  RRV.sub <- RRV.c[which(RRV.c$SA2 == snum), ]

  RRV.sub <- RRV.sub %>%

    arrange(Date)

  RRV.sub$RRVhn <- cumsum(RRV.sub$RRVn)

  RRV.f <- dplyr::bind_rows(RRV.f, RRV.sub)

  print(paste(i, RRV.sub[1, 3], sep = ","))

}

RRV <- RRV.f %>%

  group_by(Week, SA2) %>%

  summarise(Date = first(Date), HPavg = mean(value), RRVn = sum(RRVn), RRVhn = max(RRVhn))

# Calculate the susceptible human populations.

HP$HPsus <- as.numeric(as.integer(HP$HPavg - HP$RRVhn - HP$Cases))
```

Model building.

Split training set and validation set (use data of Brisbane as an example)

```
BMAA <- MAAD[MAAD$Region == "B", ]

BMAAn <- BMAA[!is.na(BMAA$Totalmosquito), ]

# Calculating CCF of lagged variables

BMAAs <- BMAAn %>%

  group_by(Year, Week) %>%

  summarise(MaxTavg = mean(MaxTavg), Rangeavg = mean(Rangeavg), MaxHavg = mean(MaxHavg), MinHavg = mean(MinHavg), Rainavg = mean(Rainavg), VP3avg = mean(VP3avg), VP9avg = mean(VP9avg), Evapavg = mean(Evapavg), Maaeavg = mean(Maaeavg), SOIavg = mean(SOIavg), Elmed = median(Elmed), Lamed = median(Lamed), NDVIavg = mean(NDVIavg), HPsus = mean(HPsus), RRVn = sum(RRVn), Aevg = mean(Ae.vigilax), Aevt = mean(Ae.vittiger), Aent = mean(Ae.notoscriptus), Cxal = mean(Cx.annulirostris), Cxst = mean(Cx.sitiens), aepc = mean(Ae.procax), anan = mean(An.annulipes), cqli = mean(Cq.linealis), cxob = mean(Cx.orbostiensis), mauf = mean(Ma.uniformis), vefu = mean(Ve.funerea), vemk = mean(Ve.marks), Tomos = mean(Totalmosquito))

RRV <- ts(BMAAs[which(BMAAs$Year > 2012 | (BMAAs$Year == 2012 & BMAAs$Week > 29)), ]$RRVn)


# Calculate CCF by spearman, predictors including mosquito abundance

cor=c()

for (Ncor in 0:52) {

  exl <- lag(BMAAs[ ,3:15], n = Ncor)

  exl1 <- merge(BMAAs[ ,1:2], exl, by = 0, all = TRUE)

  exl2 <- exl1[order(exl1$Week), ]

  exl3 <- exl2[which(exl2$Year > 2012 | (exl2$Year == 2012 & exl2$Week > 29)), 4:16]

  cors <- cor(exl3, RRV, use = "complete.obs", method = 'spearman')

  cors

  cor <- cbind(cor, cors)
```

```
}
# Get two largest (positive, negative and absolute) values for each exposure
Tcor <- t(cor)
length(Tcor[ ,1])
Mcor <- c()
for (C in 1:13) {
  M <- c()
  maxc <- which(Tcor[ ,C] == max(Tcor[ ,C]))
  minc <- which(Tcor[ ,C] == min(Tcor[ ,C]))
  M <- c(maxc[1], minc[1])
  Mcor <- rbind(Mcor, M)
}
rownames(Mcor) <- rownames(cor)
colnames(Mcor) <- c("Pos_max", "Neg_max")

# The lagged time for mosquito abundance are 3 months
cor=c()
for (Ncor in 0:13) {
  exl <- lag(BMAAs[ ,18:30], n = Ncor)
  exl1 <- merge(BMAAs[ ,1:2], exl, by = 0, all = TRUE)
  exl2 <- exl1[order(exl1$Week), ]
  exl3 <- exl2[which(exl2$Year > 2012 | (exl2$Year == 2012 & exl2$Week > 29)), 4:16]
  cors <- cor(exl3, RRV, use = "complete.obs", method = 'spearman')
  cors
  cor <- cbind(cor, cors)
}
# Get two largest (positive, negative and absolute) values for each exposure
```

```
Tcor <- t(cor)
length(Tcor[ ,1])
Ncor <- c()
for (C in 1:13) {
  N <- c()
  maxc <- which(Tcor[ ,C] == max(Tcor[ ,C]))
  minc <- which(Tcor[ ,C] == min(Tcor[ ,C]))
  N <- c(maxc[1], minc[1])
  Ncor <- rbind(Ncor, N)
}
rownames(Ncor) <- rownames(cor)
colnames(Ncor) <- c("Pos_max", "Neg_max")

# Add lagged variables to the original data set
BMAA <- BMAA %>%
  arrange(Year, Week)
BMAA.tr <- BMAA %>%
  group_by(SA2) %>%
  mutate(MaxTlag1 = dplyr::lag(MaxTavg, n = Mcor[1,1], default = NA),
         MaxTlag2 = dplyr::lag(MaxTavg, n = Mcor[1,2], default = NA),
         Rangelag1 = dplyr::lag(Rangeavg, n = Mcor[2,1], default = NA),
         Rangelag2 = dplyr::lag(Rangeavg, n = Mcor[2,2], default = NA),
         MaxHlag1 = dplyr::lag(MaxHavg, n = Mcor[3,1], default = NA),
         MaxHlag2 = dplyr::lag(MaxHavg, n = Mcor[3,2], default = NA),
         MinHlag1 = dplyr::lag(MinHavg, n = Mcor[4,1], default = NA),
         MinHlag2 = dplyr::lag(MinHavg, n = Mcor[4,2], default = NA),
         Rainlag1 = dplyr::lag(Rainavg, n = Mcor[5,1], default = NA),
```

```
Rainlag2 = dplyr::lag(Rainavg, n = Mcor[5,2], default = NA),
VP3lag1 = dplyr::lag(VP3avg, n = Mcor[6,1], default = NA),
VP3lag2 = dplyr::lag(VP3avg, n = Mcor[6,2], default = NA),
VP9lag1 = dplyr::lag(VP9avg, n = Mcor[7,1], default = NA),
VP9lag2 = dplyr::lag(VP9avg, n = Mcor[7,2], default = NA),
Evaplag1 = dplyr::lag(Evapavg, n = Mcor[8,1], default = NA),
Evaplag2 = dplyr::lag(Evapavg, n = Mcor[8,2], default = NA),
Maaelag1 = dplyr::lag(Maaeavg, n = Mcor[9,1], default = NA),
Maaelag2 = dplyr::lag(Maaeavg, n = Mcor[9,2], default = NA),
SOIlag1 = dplyr::lag(SOIavg, n = Mcor[10,1], default = NA),
SOIlag2 = dplyr::lag(SOIavg, n = Mcor[10,2], default = NA),
ELlag1 = dplyr::lag(Elmed, n = Mcor[11,1], default = NA),
ELlag2 = dplyr::lag(Elmed, n = Mcor[11,2], default = NA),
LAlag1 = dplyr::lag(Lamed, n = Mcor[12,1], default = NA),
LAlag2 = dplyr::lag(Lamed, n = Mcor[12,2], default = NA),
NDVIlag1 = dplyr::lag(NDVIavg, n = Mcor[13,1], default = NA),
NDVIlag2 = dplyr::lag(NDVIavg, n = Mcor[13,2], default = NA),
Aevglag1 = dplyr::lag(Ae.vigilax, n = Ncor[1,1], default = NA),
Aevglag2 = dplyr::lag(Ae.vigilax, n = Ncor[1,2], default = NA),
Aevtlag1 = dplyr::lag(Ae.vittiger, n = Ncor[2,1], default = NA),
Aevtlag2 = dplyr::lag(Ae.vittiger, n = Ncor[2,2], default = NA),
Aentlag1 = dplyr::lag(Ae.notoscriptus, n = Ncor[3,1], default = NA),
Aentlag2 = dplyr::lag(Ae.notoscriptus, n = Ncor[3,2], default = NA),
Cxallag1 = dplyr::lag(Cx.annulirostris, n = Ncor[4,1], default = NA),
Cxallag2 = dplyr::lag(Cx.annulirostris, n = Ncor[4,2], default = NA),
Cxstlag1 = dplyr::lag(Cx.sitiens, n = Ncor[5,1], default = NA),
Cxstlag2 = dplyr::lag(Cx.sitiens, n = Ncor[5,2], default = NA),
```

```
        Aepclag1 = dplyr::lag(Ae.procax, n = Ncor[6,1], default = NA),
        Aepclag2 = dplyr::lag(Ae.procax, n = Ncor[6,2], default = NA),
        Ananlag1 = dplyr::lag(An.annulipes, n = Ncor[7,1], default = NA),
        Ananlag2 = dplyr::lag(An.annulipes, n = Ncor[7,2], default = NA),
        Cqlilag1 = dplyr::lag(Cq.linealis, n = Ncor[8,1], default = NA),
        Cqlilag2 = dplyr::lag(Cq.linealis, n = Ncor[8,2], default = NA),
        Cxoblag1 = dplyr::lag(Cx.orbostiensis, n = Ncor[9,1], default = NA),
        Cxoblag2 = dplyr::lag(Cx.orbostiensis, n = Ncor[9,2], default = NA),
        Mauflag1 = dplyr::lag(Ma.uniformis, n = Ncor[10,1], default = NA),
        Mauflag2 = dplyr::lag(Ma.uniformis, n = Ncor[10,2], default = NA),
        Vefulag1 = dplyr::lag(Ve.funerea, n = Ncor[11,1], default = NA),
        Vefulag2 = dplyr::lag(Ve.funerea, n = Ncor[11,2], default = NA),
        Vemklag1 = dplyr::lag(Ve.marks, n = Ncor[12,1], default = NA),
        Vemklag2 = dplyr::lag(Ve.marks, n = Ncor[12,2], default = NA),
        Tomoslag1 = dplyr::lag(Totalmosquito, n = Ncor[13,1], default = NA),
        Tomoslag2 = dplyr::lag(Totalmosquito, n = Ncor[13,2], default = NA),
        RRVlag1 = dplyr::lag(RRVn, n = 1, default = NA),
        RRVlag2 = dplyr::lag(RRVn, n = 2, default = NA),
        RRVlag3 = dplyr::lag(RRVn, n = 3, default = NA),
        RRVlag4 = dplyr::lag(RRVn, n = 4, default = NA),
        RRVlag5 = dplyr::lag(RRVn, n = 5, default = NA),
        RRVlag6 = dplyr::lag(RRVn, n = 6, default = NA),
        RRVlag7 = dplyr::lag(RRVn, n = 7, default = NA),
        RRVlag8 = dplyr::lag(RRVn, n = 8, default = NA))
BMAA.tr$RRVh4 <- (BMAA.tr$RRVlag1)*4/10 + (BMAA.tr$RRVlag2)*3/10 +
(BMAA.tr$RRVlag3)*2/10 + (BMAA.tr$RRVlag4)*1/10
# Training set 1
```

```
#2014.8-2016.8

BMAA.va <- BMAA.tr[which(BMAA.tr$Year>2014 | (BMAA.tr$Year == 2014 &
BMAA.tr$Week>31)), ]

BMAA.va <- BMAA.va[which(BMAA.va$Year<2016 | (BMAA.va$Year == 2016 &
BMAA.va$Week<32)), ]

#2012.7-2014.7

BMAA.tr <- BMAA.tr[which(BMAA.tr$Year<2014 | (BMAA.tr$Year == 2014 &
BMAA.tr$Week<32)), ]

BMAA.tr <- BMAA.tr[which(BMAA.tr$Year>2012 | (BMAA.tr$Year == 2012 &
BMAA.tr$Week>29)), ]

# Training set 2

#2016.8-2018.8

BMAA.va <- BMAA.tr[which(BMAA.tr$Year>2016 | (BMAA.tr$Year == 2016 &
BMAA.tr$Week>31)),]

BMAA.va <- BMAA.va[which(BMAA.va$Year<2018 | (BMAA.va$Year == 2018 &
BMAA.va$Week<31)),]

#2012.7-2016.7

BMAA.tr <- BMAA.tr[which(BMAA.tr$Year<2016 | (BMAA.tr$Year == 2016 &
BMAA.tr$Week<32)),]

BMAA.tr <- BMAA.tr[which(BMAA.tr$Year>2012 | (BMAA.tr$Year == 2012 &
BMAA.tr$Week>29)),]

# Training set 3

#2018.8-

BMAA.va <- BMAA.tr[which(BMAA.tr$Year>2018 | (BMAA.tr$Year == 2018 &
BMAA.tr$Week>30)),]

#2012.7-2018.7

BMAA.tr <- BMAA.tr[which(BMAA.tr$Year<2018 | (BMAA.tr$Year == 2018 &
BMAA.tr$Week<31)),]

BMAA.tr <- BMAA.tr[which(BMAA.tr$Year>2012 | (BMAA.tr$Year == 2012 &
BMAA.tr$Week>29)),]
```

```
BMAA.tr <- BMAA.tr[!is.na(BMAA.tr$Totalmosquito), ]
BMAA.va <- BMAA.va[!is.na(BMAA.va$Totalmosquito), ]

BMAA.tr_nf <- BMAA.tr
BMAA.tr <- within(BMAA.tr, {
  Elmed <- as.factor(Elmed)
  Lamed <- as.factor(Lamed)
})

BMAA.va <- within(BMAA.va, {
  Elmed <- as.factor(Elmed)
  Lamed <- as.factor(Lamed)
})

Data.tr <- BMAA.tr
Data.tr_nf <- BMAA.tr_nf
Data.va <- BMAA.va

# Model building
fit.negbin <- function(variables, data) {
  a <- "RRVn ~ "
  os <- "offset(log(HPsus))"
  for (m in 1:length(unique(variables))) {
    a <- paste(a, variables[m], sep = "+")
  }
  fit=glm.nb(as.formula(paste(a, os, sep = "+")), data = data)
  return(fit)
```

```
}
pvalue <- vector()
i <- 1
for (m in 1:length(unique(expt))) {
  fit.rep <- fit.negbin(expt[m], Data.tr_nf)
  pvalue.rep <- summary(fit.rep)$coefficients[ ,4]
  pvalue <- c(pvalue, pvalue.rep[-1])
  print(i)
  i = i + 1
}
pvalue <- as.data.frame(pvalue)
index <- pvalue$pvalue > 0.1
rvar.p <- vector()
if(any(pvalue$pvalue > 0.1))
  rvar.p <- rownames(pvalue)[row(pvalue) * index]
rvar.p
if(length(rvar.p) > 0) {
  np <- match(rvar.p, expt)
  np <- np[!is.na(np)]
  expt <- expt[-c(np)]
} else {
  expt <- expt
}
# Standardized regression coefficients
fit.negbin.stcoef <- function(variables, data) {
  stcoef <- vector()
  os <- "offset(log(HPsus))"
```

```
  for (m in 1:length(unique(expt))) {
    a <- "RRVn ~ scale("
    a <- paste(a, expt[m], ")", sep = "")
    fit.rep <- glm.nb(as.formula(paste(a, os, sep="+")), data = Data.tr_nf)
    coef.rep <- round(fit.rep$coefficients, digits = 2)
    stcoef <- cbind(stcoef, coef.rep[-1])
    colnames(stcoef)[m] <- expt[m]
  }
  return(stcoef)
}
stcoef <- fit.negbin.stcoef(expt, Data.tr_nf)
index <- abs(stcoef[1, ]) < 0.1
rvar.src <- vector()
if(any(abs(stcoef[1, ]) < 0.1))
  rvar.src <- colnames(stcoef)[col(stcoef) * index]
rvar.src
if(length(rvar.src) > 0) {
  expt <- expt[-c(match(rvar.src, expt))]
} else {
  expt <- expt
}
# Removed variables at step 2
rvar.s2 <- unique(c(rvar.p, rvar.src))

# Backward screen
fit.formula <- function(variables) {
  a <- "RRVn ~ "
```

```
  for (m in 1:length(unique(variables))) {
    a <- paste(a, variables[m], sep = "+")
  }
  return(a)
}
# Function of calculating VIF/BIC by removing/including exposures one by one
Selectexp.IC <- function(input, expset, s) {
  for (m in 1:length(unique(expset))) {
    a <- fit.formula(input)
    formula.rep <- as.formula(paste(paste(a, expset[m], sep = s)," + offset(log(HPsus))", sep = ""))
    fit.rep <- glm.nb(formula.rep, data = Data.tr)
    if (length(input) > 2) {
      vif.rep <- max(vif(fit.rep))
    } else {
      vif.rep <- 0
    }
    BIC.rep <- BIC(fit.rep)
    BIC.chgp <- 100 * (BIC.rep - BIC) / BIC
    IC.rep <- c(vif.rep, BIC.rep, BIC.chgp)
    IC <- rbind(IC, IC.rep)
  }
  colnames(IC) <- c("max_VIF", "BIC", "BIC_change")
  rownames(IC) <- c(expset)
  IC <- as.data.frame(IC)
  return(IC)
}
```

```
nrep <- 1

repeat {

  fit <- fit.negbin(expt, Data.tr)

  predictions <- fit %>% predict(Data.tr, type = "response")

  AIC <- AIC(fit, k = 2)

  BIC <- BIC(fit)

  HQIC <- as.numeric(-2*logLik(fit) + (length(fit$coefficients) * log(log(nrow(Data.tr)))))

  if (length(expt) > 1) {

    v <- vif(fit)

  } else {

    v <- data.frame(0, 0, 0)

  }

  v<-data.frame(v)

  index <- v$v >5

  rvar.vif <- vector()

  if(any(v[ ,1] > 5))

    rvar.vif <- rownames(v)[row(v) * index]

  rvar.vif

  if (length(rvar.vif) == 0 | length(rvar.vif) == length(expt)) {

    expti <- expt

  } else {

    expti <- expt[-c(match(rvar.vif, expt))]

  }

  # Remove variables if BIC increment is negative or lowest when removing the variable from the
model

  fit <- fit.negbin(expti, Data.tr)

  AIC <- AIC(fit, k = 2)
```

```
BIC.o <- BIC(fit)
# Remove variables with lowest BIC increment until total BIC increment reaches 1%
BIC.chg <- 0
BIC <- BIC.o
while (BIC.chg < 0.5 & length(expti) > 1) {
  IC <- c()
  IC <- Selectexp.IC(expti, expti, "-")
  rvar.ic <- rownames(IC[which(IC[ ,3] < 0), ])
  if (length(rvar.ic) == length(expti)) {
    break
  } else if (length(rvar.ic) == 0) {
    rvar.ic <- rownames(IC[which.min(IC[ ,3]), ])
  } else {
    rvar.ic <- rvar.ic
  }
  expti <- expti[-c(match(rvar.ic, expti))]
  fit <- fit.negbin(expti, Data.tr)
  BIC <- BIC(fit)
  BIC.chg <- 100 * (BIC - BIC.o) / BIC.o
}
if(length(expti) > 0) {
  rvar.ic <- expt[-c(match(expti, expt))]
} else {
  rvar.ic <- vector()
}
rvar.bs <- unique(c(rvar.vif, rvar.ic))
```

```
# Step 4 Backward Screen
 if (length(rvar.bs) == 0) {
   ivar <- expt
   exptb <- c()
   IC <- rbind(c(0, 0, 0), c(0, 0, 0))
 } else {
   if(length(rvar.bs) == length(expt)) {
     IC <- c()
     a <- c()
     IC <- Selectexp.IC("", expt, "")
     ivar <- rownames(IC[which.max(IC[,2]),])
     ivar
     exptb <- expt[-c(match(ivar, expt))]
   }
   else {
     ivar <- expt[-c(match(rvar.bs, expt))]
     exptb <- rvar.bs
   }
   fit <- fit.negbin(ivar, Data.tr)
   BIC <- BIC(fit)
   IC <- c()
   IC <- Selectexp.IC(ivar, exptb, "+")
   index <- (IC[ ,1] < 10 & IC[ ,3] < -0.1)
   ivar.bs <- vector()
   if(any(index))
     ivar.bs <- rownames(IC)[(row(IC) * index)[ ,1]]
   ivar <- c(ivar, ivar.bs)
```

```
  }

  nrep <- nrep + 1
  if ((length(ivar) == 0) | (length(ivar) == length(expt)) | (nrep > 11)) {
    break
  } else {
    expt <- ivar
  }
}

# Final check and validation
fit <- fit.negbin(expt, Data.tr)
if (length(expt) > 1) {
  v <- vif(fit)
} else {
  v <- data.frame(0, 0, 0)
}
v<-data.frame(v)
index <- v$v >5
rvar.vif <- vector()
if(any(v[ ,1] > 5))
  rvar.vif <- rownames(v)[row(v) * index]
rvar.vif
if (length(rvar.vif) == length(expt)) {
  expt <- c("Week")
  out.rep <- paste("No variables included Fainal Step", " VIF", sep = "")
  Val.out[nrow(Val.out) + 1, ] <- out.rep
```

```
} else if (length(rvar.vif) > 0) {
  expt <- expt[-c(match(rvar.vif, expt))]
} else {
  expt <- expt
}
```

Figure generation.

```
# Do prediction (use Brisbane data as an example)
fit <- fit.negbin(expt, Data.tr)
summary(fit)
predictions <- fit %>%
  predict(Data.tr, type = "response")
AIC <- AIC(fit, k = 2)
BIC <- BIC(fit)
HQIC <- as.numeric(-2 * logLik(fit) + (length(fit$coefficients) * log(log(nrow(Data.tr)))))
predB <- fit %>%
  predict(Data.tr, type = "response")
predB_v <- fit %>%
  predict(Data.va, type = "response")
predB[is.na(predB)] <- 0
predB_v[is.na(predB_v)] <- 0

T_pred <- Data.tr[c(1:3, 18:19)]
T_pred <- left_join(T_pred, DateW, by = c("Year", "Week"))
T_pred <- cbind(T_pred, predB = predB)
V_pred <- Data.va[c(1:3, 18:19)]
V_pred <- left_join(V_pred, DateW, by = c("Year", "Week"))
```

```
V_pred <- cbind(V_pred, predB_v = predB_v)
T_pred <- as.data.frame(T_pred)
V_pred <- as.data.frame(V_pred)

V_pred = rename(V_pred, predB = predB_v)
All_pred <- rbind(T_pred, V_pred)

All_predB <- All_pred %>%
  group_by(Week, Date, Year) %>%
  summarise(RRVn = sum(RRVn), predB = sum(predB))

# Plot predictive curve (use Brisbane data as an example)
fig <- ggplot(data = All_predB, aes(Date)) +
  geom_line(aes(y = RRVn, color = "Real data")) +
  geom_line(aes(y = predB, color = "Predicted value")) +
  scale_colour_manual(values = c("slateblue3", "black")) +
  geom_vline(aes(xintercept = as.Date("2018-01-30")), color = "orange3")+
  annotate(geom = "text", x = as.Date("2017-10-30"), y = 60, size = 5, color = "orange3",
       label = "Training Set                    Validation Set") +
  labs(x = "", y = "RRV cases") +
  scale_x_date(date_labels = "%Y", date_breaks = "1 year", minor_breaks = "1 week", expand =
c(0, 0)) +
  theme(text = element_text(size = 12),
      legend.title = element_blank(),
      legend.position = c(0.2, 0.75),
      axis.line = element_line(colour = "grey20", size = 0.2),
      axis.text.x = element_text(angle = 25, vjust = 0.7),
```

```
      axis.title.x = element_blank (),
      panel.background = element_rect(fill = "white"))

# Plot mosquito abundance and log transformed RRV cases (use Brisbane data as an example)
BMAA.sp <- BMAA[which(BMAA$Year>2012 | (BMAA$Year == 2012 & BMAA$Week>29)), ]
#2012.7-
BMAA.sp <- BMAA.sp[!is.na(BMAA.sp$Totalmosquito), ]
BMAA.sp <- left_join(BMAA.sp, DateW, by = c("Year", "Week"))
BMAA.sp <- BMAA.sp %>%
  group_by(Date) %>%
  summarise(RRVn = sum(RRVn), Total = sum(Totalmosquito), Ae.vigilax = sum(Ae.vigilax),
Ae.vittiger = sum(Ae.vittiger), Ae.notoscriptus = sum(Ae.notoscriptus), Cx.annulirostris =
sum(Cx.annulirostris))
BMAA.s <- melt(BMAA.sp[-c(2)], id = "Date")
BMAA.sp$LogRRV <- log(BMAA.sp$RRVn + 1)

coeff <- 0.003 # Value used to transform the data
p <- ggplot(data = BMAA.sp, aes(x = Date, y = Total)) +
  geom_line(size = 0.7, color = "#E41A1C") +
  geom_line(aes(y = LogRRV / coeff), size = 0.8, color = "#377EB8") +
  scale_x_date(date_labels = "%Y", date_breaks = "1 year", minor_breaks ="1 week", expand =
c(0, 0)) +
  scale_y_continuous(
    name = "Total mosquito abundance",
    sec.axis = sec_axis(~. * coeff, name = "Log transformed RRV cases")
  ) +
  theme(text = element_text(size = 12),
      legend.title = element_blank(),
```

```
        legend.text = element_text(face = "italic", size = 12),
        legend.position = "top",
        axis.line = element_line(colour = "grey20", size = 0.2),
        axis.title.x = element_blank(),
        axis.text.x = element_text(angle = 25, vjust = 0.7),
        axis.title.y = element_text(size = 9, color = "#E41A1C"),
        axis.title.y.right = element_text(color = "#377EB8", size = 9),
        panel.background = element_rect(fill = "white")) +
  ggtitle("        Brisbane")

# CCF plot (use Brisbane data as an example)
BMAAs <- BMAA %>%
  group_by(Year, Week) %>%
  summarise(RRVn = sum(RRVn), Aevg = mean(Ae.vigilax), Aevt = mean(Ae.vittiger), Aent = mean(Ae.notoscriptus), Cxal = mean(Cx.annulirostris), Tomos = mean(Totalmosquito))
RRV <- ts(BMAAs$RRVn)
Bcor = c()
for (Ncor in 0:13) {
  exl <- lag(BMAAs[ ,4:8], n = Ncor)
  exl1 <- merge(BMAAs[ ,1:2], exl, by = 0, all = TRUE)
  exl2 <- exl1[order(exl1$Week), ]
  exl3 <- exl2[ ,4:8]
  Bcors <- cor(exl3, RRV, use = "complete.obs", method = 'spearman')
  Bcors
  Bcor <- cbind(Bcor, Bcors)
}
Bcor <- as.data.frame(Bcor)
```

```
row.names(Bcor) <- c("Ae.vigilax", "Ae.vittiger", "Ae.notoscriptus", "Cx.annulirostris", "Total mosquito abundance")

Bcor$Mos <- c(row.names(Rcor))

Bcor.m <- melt(Bcor, id = "Mos", variable.name = "Lag", value.name = "Correlation")

pBcor <- ggplot(data = Bcor.m, aes(x = Lag, y = Correlation, color = Mos)) +
  geom_point(size = 2) +
  geom_line(size = 0.5) +
  labs(x = "Number of lags", y = "Spearman correlation") +
  theme(text = element_text(size = 12),
        legend.title = element_blank(),
        legend.text = element_text(face = "italic", size = 12),
        legend.position = "right",
        axis.line = element_line(colour = "grey20", size = 0.2),
        axis.title.y = element_text(size = 12),
        axis.title.x = element_blank(),
        panel.background = element_rect(fill = "white")) +
  ggtitle("          Brisbane")
pB <- pBcor +
  geom_hline(aes(yintercept = 0))


# Correlation plot (use Brisbane data as an example)
B.c <- cor(BMAA[c(5:8, 19)], use = "complete.obs", method = 'spearman')
row.names(B.c)[5] <- c("Total mosquito abundance")
colnames(B.c)[5] <- c("Total mosquito abundance")
pBc <- ggcorrplot(B.c, method = "circle", lab = TRUE,
                  colors = c("#377EB8", "white", "#E41A1C")) +
  theme(text = element_text(size = 12),
```

```
    legend.title = element_blank(),
    panel.background = element_rect(fill = "white")) +
ggtitle("        Brisbane")
```
