# Supplementary figures and images for: Prediction of Ross River Virus Incidence Using Mosquito Data in Three Cities of Queensland, Australia
Source: Biology (Basel). 2023 Nov 13;12(11):1429. doi: 10.3390/biology12111429 (PMC10669834; doi:10.3390/biology12111429)

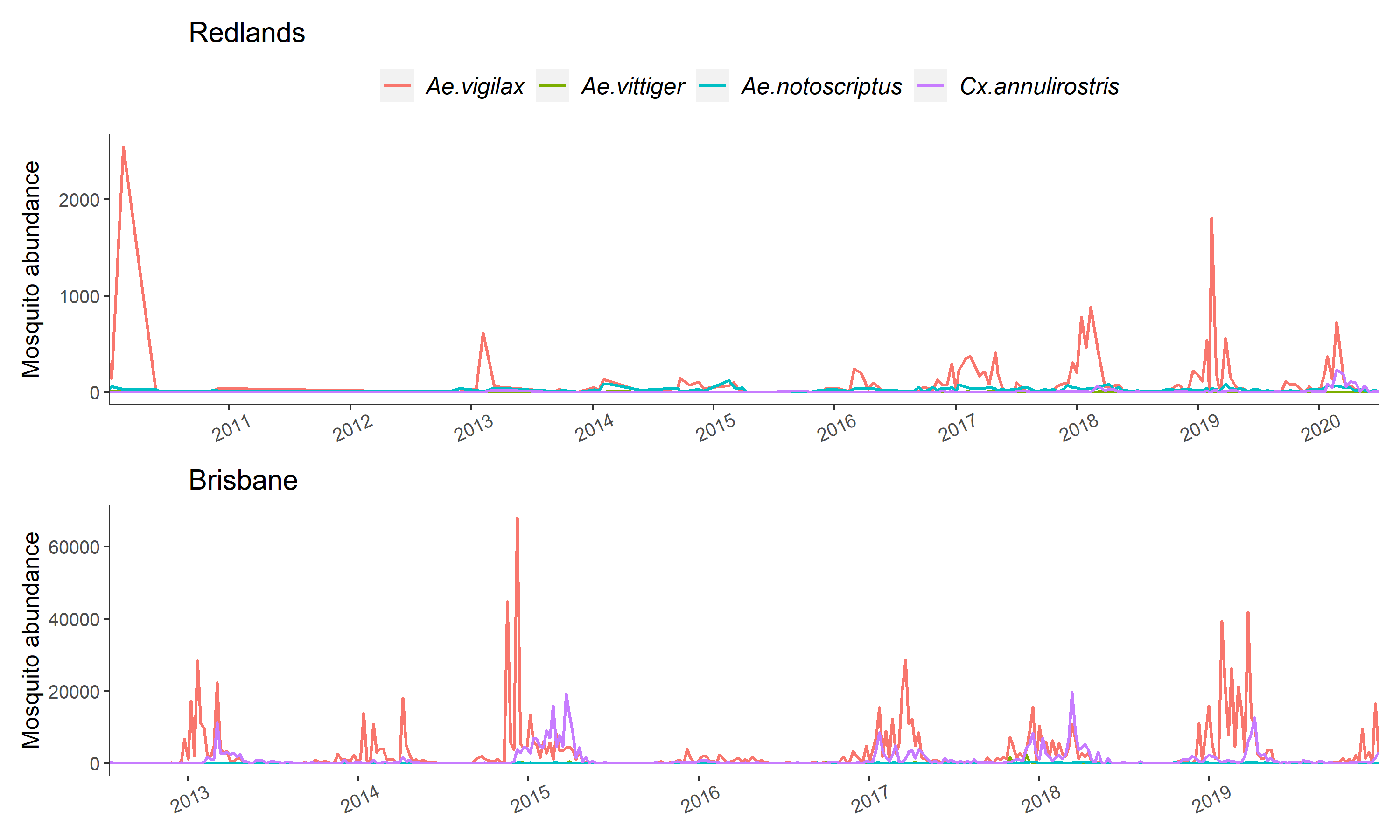

Supplement: Supplementary file 1 [file biology-12-01429-s001.zip › Supplementary Figure S1.tiff]

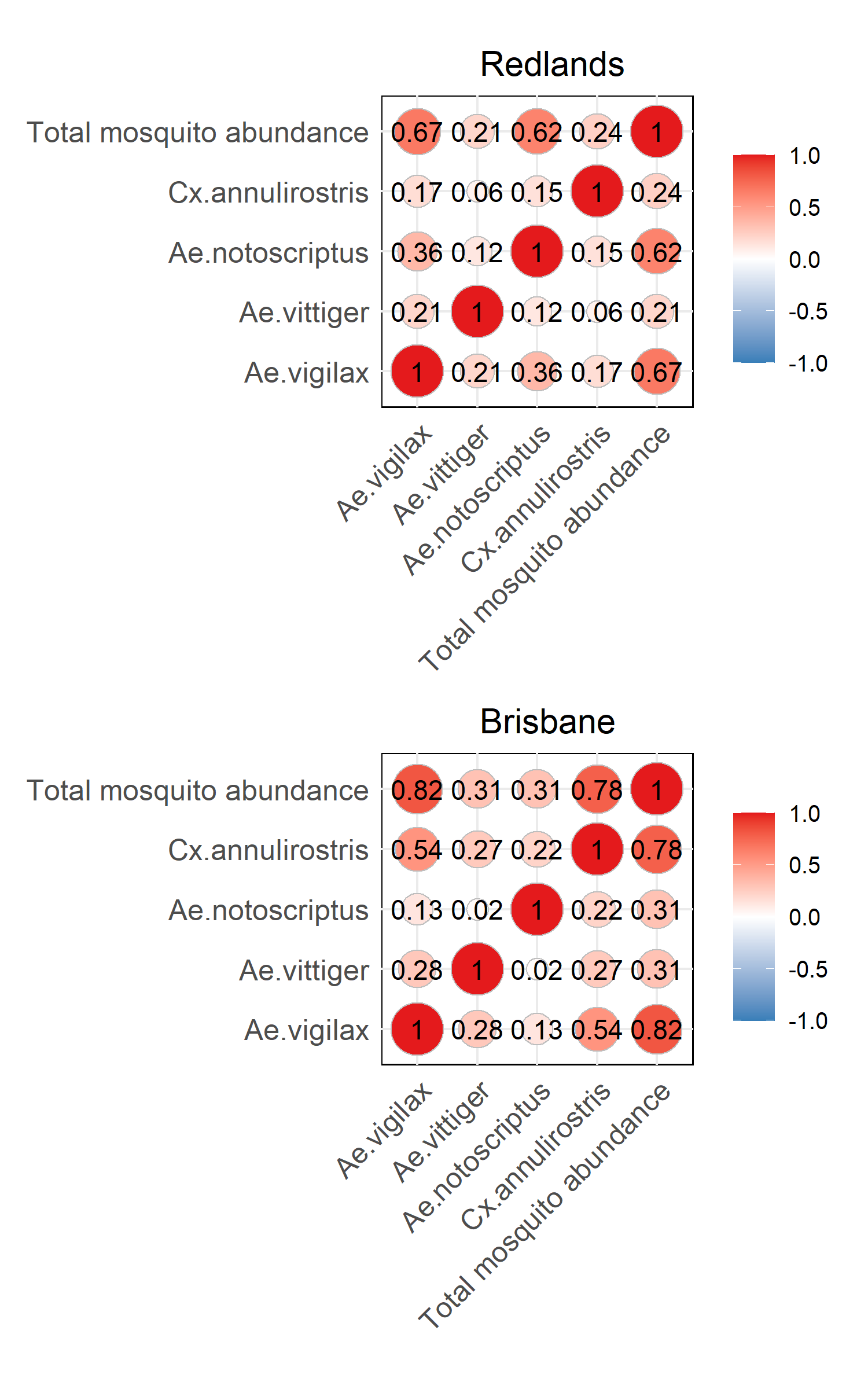

Supplement: Supplementary file 1 [file biology-12-01429-s001.zip › Supplementary Figure S2.tiff]
